# MULTIMORBIDITY, MUSCLE STRENGTH, AND FALLS AMONG OLDER MEXICAN AMERICANS

**DOI:** 10.1093/geroni/igae098.3456

**Published:** 2024-12-31

**Authors:** Alondra Uribe, Soham Al Snih

**Affiliations:** The University of Texas Medical Branch, Galveston, Texas, United States; University of Texas Medical Branch in Galveston, Galveston, Texas, United States

## Abstract

**Abstract objectives:**

To determine the relationship of multimorbidity and muscle strength with falls among older Mexican Americans without a history of falls at baseline. Design: Longitudinal study. Setting and Participants: This 12-year prospective cohort study included 899 non-institutionalized Mexican Americans aged ≥75 residing in Arizona, California, Colorado, New Mexico, and Texas from the Hispanic Established Population for the Epidemiologic Study of the Elderly.

**Methods:**

Measures include socio-demographics, medical conditions, Body Mass Index, disability, handgrip strength (HGS), depressive symptoms, pain, cognitive function, and multimorbidity (≥2 self-reported medical conditions). Participants at baseline were divided into four groups: high HGS and multimorbidity (N=349), low HGS and multimorbidity (N=263), high HGS and without multimorbidity (N=181), and low HGS and without multimorbidity (N=104). Generalized estimating equation models estimated the odds ratio (OR) and 95% Confidence Interval (CI) of falls as a function of multimorbidity and HGS groups controlling for all covariates.

**Results:**

The OR of falls was 0.72 (CI=0.54-0.95) for those with multimorbidity and high HGS, 0.52 (95% CI=0.33-0.82) for those without multimorbidity and high HGS, and 0.46 (95% CI=0.29-0.74) for those without multimorbidity and low HGS, versus those with multimorbidity and low HGS, after controlling for all covariates. Conclusions and Implications: Mexican American older adults with multimorbidity and high HGS had a 28% decreased risk of falls over time. Increasing muscle strength through exercise may help prevent falls among those with multimorbidity.

